# Paid Sick Leave and Sickness Benefits for employees’ economic and job security: A Scoping Review: Yewon Kwon

**DOI:** 10.1093/eurpub/ckac131.550

**Published:** 2022-10-25

**Authors:** Y Kwon, H Lee, K Ryu, D Moon, H Chung

**Affiliations:** 1 Department of Public Health Sciences, Graduate School, Korea University, Seoul, South Korea; 2 BK21 FOUR Learning Health Systems, Korea University, Seoul, South Korea; 3 People’s Health Institute, Seoul, South Korea; 4 School of Health Policy and Management, Korea University, Seoul, South Korea

## Abstract

In health emergencies, such as in the COVID-19 pandemic, the need to expand or introduce the Paid sick leave(PSL) and Sickness benefits(SB) increases. They are key components of the universal health coverage(UHC) and active labor market policies(ALMPs) that enable workers to take care of their health and guarantee return-to-work after recovery. This study examines effects those policies in achieving economic stability and job security of covered workers through a scoping review. Studies were selected using the search terms ‘paid sick leave', ‘sickness benefits', ‘paid sick day', and ‘earned sick leave’ in PubMed and Web of Science. Our search conducted on 6th April 2021 yielded 1,030 articles, of which 22 articles were included in the review. All articles were analyzed by the 4 sub-groups(employees, families, employers, and government) and we investigated indicators of socio-economic impacts on their lives. Articles are largely PSL(90.9%)-focused. PSL guarantees not only workers’ job security by securing employment agreement, but also their income security by promising part of wages enough to afford healthcare and living expenses during the medical treatment and recovery. Additionally, PSL attenuates employers’ financial risk, as it reduces presenteeism while increasing the return-to-work rate. Moreover, PSL and SB reduce the total healthcare and social security expenditures of the government. To sum up, PSL and SB guarantee health and labor rights by ensuring income and job security to employees while assuring financial stability to both employers, and the government. However, as the previous studies paid less attention on the equity of these impacts at the system levels, future research should more focus on the dimension.

**Key messages:**

• PSL and SB guarantee health and labour rights by ensuring income and job security for employees, while assuring financial stability for both employers and the government.

• The previous studies that examined the effects of PSL and SB paid less attention on the equity of ensuring income and employment security, therefore future studies should focus more on this dimension.

